# Influence of the *Potato leafroll virus* and virus-infected plants on the arrestment of the aphid, Myzus persicae

**DOI:** 10.1673/2006_06_22.1

**Published:** 2006-09-28

**Authors:** Thomas M. Mowry, John D. Ophus

**Affiliations:** Department of Plant, Soil and Entomological Sciences, University of Idaho, Parma Research and Extension Center, 29603 U of I Lane, Parma, ID 83660-6699 USA

**Keywords:** *Potato leafroll virus* PLRV

## Abstract

A series of experiments was conducted using membrane sachets containing MP148 diet or phosphate-buffered sucrose with and without purified Potato leafroll virus to determine if direct encounter with the virus would arrest the aphid, Myzus persicae (Sulzer) (Homoptera:Aphididae). In only two out of 36 tests were there significantly more aphids settled on sachets containing the virus. In all other tests, there were either significantly fewer aphids on sachets containing virus or there were no differences between virus treatments and control sachets without virus. In an experiment using excised Physalis floridana leaves, twice as many M. persicae settled on virus-infected leaves as on noninfected control leaves. Taken together, the results indicate that arrestment of M. persicae on potato leaf roll virus-infected plants may be due to enhanced nutritional qualities resulting from disease, but not from direct encounter with or detection of the virus.

## Introduction

 Potato leafroll virus (PLRV) is a luteovirus (Harrison 1984; Thomas 1987) that is transmitted in a persistent, circulative, nonpropagative manner by all its aphid vectors (Nault 1997; Sylvester 1980), of which Myzus persicae (Sulzer) is the most efficient and economically important. This mechanism of transmission requires complex biochemical/physiological interactions between PLRV and at least three aphid vector tissues. In two of these tissues, midgut epithelium and the accessory salivary gland, it is probable that receptor-mediated endocytosis results in acquisition, following viral uptake from an infected plant, and subsequent vector infectivity, respectively (Garret et al. 1993, 1996; Gildow 1982, 1993). Between these transcellular events, PLRV moves passively through the hemocoel, presumably protected from proteolytic degradation through interaction with the endosymbiotic protein symbionin (Hogenhout et al. 1998; van den Heuvel et al. 1994). If, and how, PLRV might interact with other tissues within M. persicae, or if there is any sensory feedback from virus-tissue interactions, is unknown.

Diseased plants, resulting from viral infection, often affect the performance of aphids that transmit the respective viruses. Aphid vectors that fed on plants infected with certain strains of barley yellow dwarf virus had higher fecundities (Fereres et al. 1989) and produced more alatae (Gildow 1980, 1983) than those that fed on noninfected plants. Hodgson (1981) reported that M. persicae fed on turnips infected with Turnip mosaic virus had higher live body weights than those fed on noninfected plants. M. persicae reared on PLRV-infected potatoes had significantly greater mean growth rates and intrinsic rates of increase than those reared on potatoes infected with other viruses not transmitted in a circulative manner or on noninfected potatoes (Castle and Berger 1993). These apparent benefits to aphid vectors are thought to be based on enhanced nutritional quality of infected plants due to nitrogen metabolism (Gildow 1980, 1983) and/or the accumulation of carbohydrates (Sylvester 1987; Thomas 1987).

The complex interactions between PLRV and M. persicae coupled with the apparent benefits of feeding on infected plants suggested the hypothesis that direct detection of the virus during the circulative transmission process might arrest aphids on virus-infected plants, apart from the putative nutritional influence. We speculated that this might be especially important in the very common situation in which mid- to late-season PLRV infection of potatoes results in relatively high virus titers, but no observable disease symptoms that might influence arrestment. Therefore, a series of simple experiments was conducted to determine if direct encounter with PLRV would arrest M. persicae at the viral acquisition source.

## Materials and Methods

A symptomatically severe PLRV isolate (LR-7), originally provided by P. E. Thomas, USDA-ARS, Prosser, WA, USA, was maintained in Physalis floridana Rydb. by aphid transfer. LR-7 was purified from infected Datura stramonium L. by P. H. Berger, Division of Plant Pathology, University of Idaho, Moscow, ID, USA, using the method of D’Arcy et al. 1989 and the final viral pellet was suspended in citrate buffer (0.1 M Na_3_C_6_H_5_O_7_·2H_2_O, 0.01 M EDTA, pH 6.4).

Two M. persicae clones were used in all experiments. Clone OUR transmits PLRV very efficiently and was collected from potato in the field. It has been in laboratory culture for approximately 25 years. Clone BP23 transmits PLRV less efficiently and was collected from an infested green pepper plant found during a bedding plant survey conducted in 1992 (Halbert and Mowry 1992). Virus-free aphid colonies were maintained on Indian mustard, Brassica juncea L., cv. Florida Broadleaf, in an insectary room at 22 ± 2°C, 40–60% r.h., and 16:8 LD.

**Figure 1:**
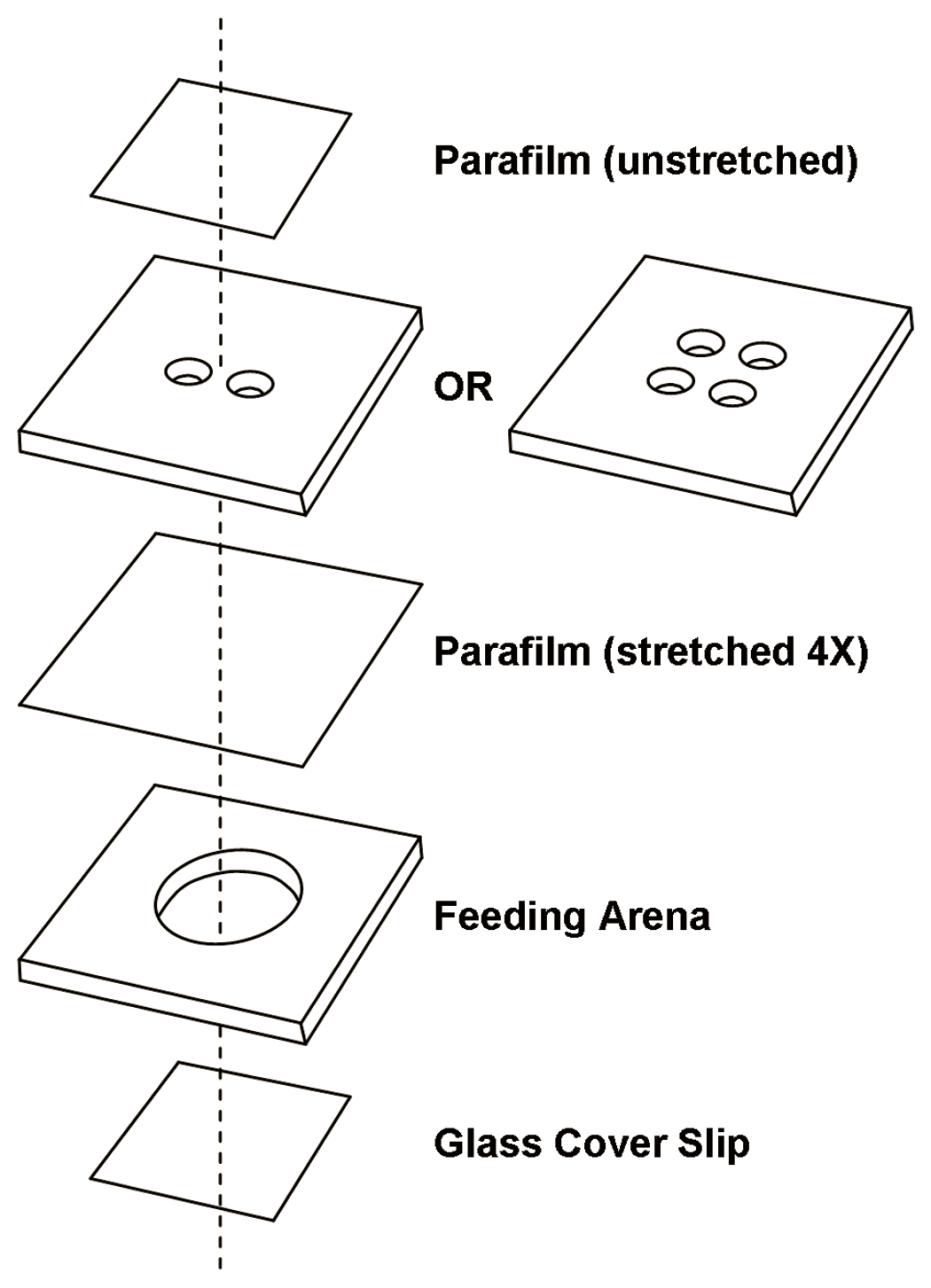
Diagram of the feeding chambers used in dual or multiple treatment experiments. See text for dimensions.

Purified PLRV at concentrations of 25, 50, and 100 μg/ml were prepared in two media and incorporated into Parafilm^®^ membrane sachets. MP148 diet (Harrewijn 1983) has been used for PLRV acquisition experiments (van den Heuvel et al. 1994). Phosphate-buffered sucrose (0.05 M Na_2_HPO_4_, pH 6.8, 15% sucrose) has been used in aphid feeding experiments (Berlandier 1996) and lacks the nutrients contained in MP148 that may mask the possible arrestment effects of PLRV. Controls containing no PLRV were prepared by adding the same volumes of virus-free citrate buffer that corresponded to the volumes used to prepare the viral treatments. Two different types of feeding chambers were constructed to perform dual and multiple treatment experiments (Figure 1). For dual treatment experiments, chambers were constructed using two, 40 mm-square pieces of 3.2 mm-thick, low density, translucent-white polyethylene sandwiched together. To form the feeding arena, a 19 mm diameter hole was drilled in the center of the lower piece of polyethylene and covered with a 22 mm-square glass cover slip. The upper piece of polyethylene had two, 8 mm-diameter holes, spaced 2 mm apart, drilled in the center to form the reservoirs for membrane sachets. A 40 mm-square piece of Parafilm^®^ was stretched to approximately four times its original size and pressed over one surface of the polyethylene, covering the holes and forming two sachets. Sixty μl of 25, 50, or 100 μg/ml PLRV in MP148 diet or phosphate-buffered sucrose was pipetted into one sachet and 60 μl of the appropriate medium control were pipetted into the other. A second piece of unstretched Parafilm^®^ was placed over the top of the sachets to prevent evaporation. Fifteen second and third instar M. persicae from either clone were placed in the feeding arena which was immediately covered with the sachets. The chambers were placed on a wire rack and held in an insectary room under the same conditions as for the clonal M. persicae cultures. After 24 h, the numbers of aphids settled on the PLRV and control sachets, as well as those not settled, were recorded by sliding a mirror under the wire rack so as not to disturb the chambers. All dual-treatment experiments were set up as randomized complete block designs with six replications and incorporating one PLRV concentration per chamber. Three chambers individually incorporating 25, 50, or 100 μg/ml PLRV constituted a complete replication. Data were subjected to analyses of variance (ANOVA) and, when the ANOVA results were significant, to Tukey’s HSD mean separation test (Wilkinson 1997). To meet the assumption of equal variance, raw data from some experiments were transformed to log_10_(aphids + 1) (Snedecor & Cochran 1989).

For multiple-treatment experiments, the protocol was identical to the dual-treatment experiments, except that 10 aphids were placed in the feeding arena. The sachet half of the feeding chambers had four, 8 mm-diameter holes, drilled in a square pattern, into which was pipetted 60 μl of 0, 25, 50, and 100 μg/ml PLRV in MP148 diet or phosphate-buffered sucrose. The chambers were held and data recorded as in the dual-treatment experiments. All multiple-treatment experiments were set up as randomized complete block designs with 10 replications and each chamber constituted a complete replication. Data were analyzed as above.

For comparative purposes, an experiment was conducted using leaves excised from PLRV-infected P. floridana and noninfected control plants. The feeding arena was constructed in the same manner as in the sachet experiments and 15 second and third instar M. persicae from either clone were placed in each arena. Two P. floridana leaves (one PLRV-infected and one noninfected), showing no signs of PLRV infection or natural senescence, were placed over the arena opening, with each leaf covering approximately one half of the arena diameter. The leaves were held to the arena with double-sided tape. The petioles of both leaves were inserted through a foam rubber plug into a glass shell vial filled with deionized water to keep the leaves turgid. A piece of polyethylene, covered on one side with black construction paper, was placed over the top of the leaves to eliminate any shadows that might be caused by the overlapping leaves and to reduce color differences that might have attracted the aphids toward one or the other of the leaves. As with the sachet experiments, 24 h after the aphids were placed in the feeding chamber their locations were recorded. This experiment was set up as a randomized complete block design with six replications with each chamber constituting a replication. Data were analyzed as above.

## Results

In the first dual-treatment experiment in which MP148 diet served as the sachet medium for clone OUR, the mean number of aphids settled on the two treatments or not settled differed significantly in the 25 μg/ml PLRV test (*F* = 12.31, df = 2, *P* = 0.0007) and in the 50 μg/ml test (*F* = 7.31, df = 2, *P* = 0.0061) after 24 h (Table 1). According to Tukey’s HSD test, significantly more aphids were settled on the 25 μg/ml PLRV sachet (*P* = 0.0005) and the 50 μg/ml PLRV sachet (*P* = 0.0056) than on their respective controls. However, there was no significant difference in the mean number of aphids settled on the two treatments or not settled in the 100 μg/ml test (*F* = 2.32, df = 2, *P* = 0.1330). For clone BP23, there were significant differences in aphid settling responses for the 25 μg/ml PLRV test (*F* = 21.00, df = 2, *P* < 0.0001), the 50 μg/ml PLRV test (*F* = 42.63, df = 2, *P* < 0.0001), and the 100 μg/ml PLRV test (*F* = 22.01, df = 2, *P* < 0.0001) after 24 h (Table 1). Tukey’s HSD test indicated that the significance found in the ANOVAs was attributable wholly to aphids not settled and there were no significant differences between the two sachet treatments.

**Table 1:**
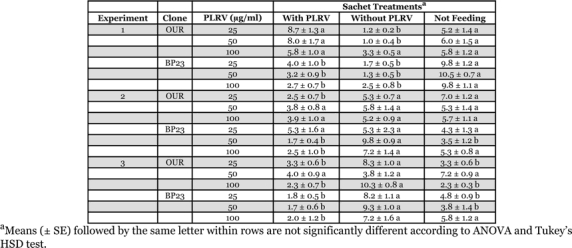
Arrestment of Myzus persicae on Parafilm^®^ membrane sachets containing MP148 aphid diet with and without purified Potato leafroll virus. The experiments were conducted as dual-treatment (two sachets) tests with one sachet containing the respective amount of virus and the other containing only diet.

In the second dual-treatment experiment in which MP148 diet served as the sachet medium for clone OUR, the mean number of aphids settled on the two treatments or not settled differed significantly in the 25 μg/ml PLRV test (*F* = 6.97, df = 2, *P* = 0.0002) after 24 h (Table 1). Tukey’s HSD test revealed that there were significantly more aphids settled on the control sachet (*P* = 0.0058) than on the 25 μg/ml PLRV sachet. There were no significant differences in the mean number of aphids settled on the two treatments or not settled in the 50 μg/ml PLRV test (*F* = 0.69, df = 2, *P* = 0.5161) or the 100 μg/ml PLRV test (*F* = 0.87, df = 2, *P* = 0.4394). For clone BP23, there was no significant difference in the mean number of aphids settled on the two treatments or not settled in the 25 μg/ml PLRV test (*F* = 0.08, df = 2, *P* = 0.9246) after 24 h (Table 1). However, the mean number of aphids settled on the two treatments or not settled differed significantly in the 50 μg/ml PLRV test (*F* = 21.28, df = 2, *P* < 0.0001) and the 100 μg/ml PLRV test (*F* = 4.46, df = 2, *P* = 0.0303). Tukey’s HSD test revealed significantly more aphids settled on the control sachets than on the respective PLRV sachets in the 50 (*P* = 0.0001) and 100 (*P* = 0.0248) μg/ml PLRV tests (Table 1).

In the third dual-treatment experiment using MP148 diet for clone OUR, the mean number of aphids settled on the two treatments or not settled differed significantly in the 25 μg/ml PLRV test (*F* = 15.63, df = 2, *P* = 0.0002) and Tukey’s HSD test revealed that this was attributable to more aphids settled on the control sachet (*P* = 0.0006; Table 1). There was no significant difference in the mean number of aphids in any situation in the 50 μg/ml PLRV test (*F* = 3.25, df = 2, *P* = 0.0672). There was a significant difference in the mean number of aphids settled or not settled in the 100 μg/ml PLRV test (*F* = 56.47, df = 2, *P* < 0.0001) and Tukey’s HSD test again revealed that this was due to more aphids settled on the control sachet (P < 0.0000; Table 1). For clone BP23, there were significant differences in the mean number of aphids between treatments in the 25 (*F* = 14.01, df = 2, *P* = 0.0004), 50 (F = 14.72, df = 2, *P* = 0.0003), and 100 (*F* = 4.92, df = 2, *P* = 0.0228) μg/ml PLRV tests after 24 h (Table 1). Tukey’s HSD test revealed that significantly more aphids were settled on the control sachets than on the PLRV sachets for all treatments (*P* = 0.0003, *P* = 0.0003, and *P* = 0.0221, respectively).

In the first multiple-treatment experiment with MP148 diet as the sachet medium, the mean number of aphids settled on the sachets or not settled differed significantly in the test with clone OUR (*F* = 42.26, df = 4, *P* < 0.0001) and in the test with clone BP23 (*F* = 7.26, df = 4, *P* = 0.0001) after 24 h (Table 2). Tukey’s HSD test revealed that the significance found in the ANOVAs was attributable to those aphids not settled and there were no significant differences between any sachet treatments. In the second multiple-treatment experiment, the mean number of aphids differed significantly in the test with clone OUR (*F* = 4.68, df = 4, *P* = 0.0030), but Tukey’s HSD test showed that this was due wholly to those aphids not settled on any sachet (Table 2). In the test with clone BP23, there were no significant differences in the mean number of aphids in any situation (*F* = 1.73, df = 4, *P* = 0.1597).

**Table 2:**
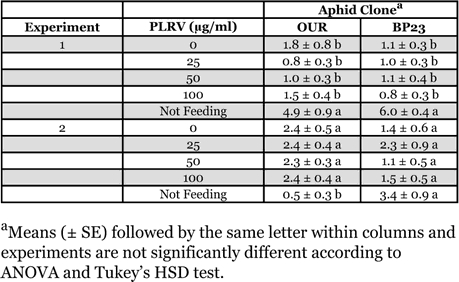
Arrestment of Myzus persicae on Parafilm^®^ membrane sachets containing MP148 aphid diet with various concentrations of purified Potato leafroll virus. The experiments were conducted as multiple-treatment tests with all virus concentrations presented simultaneously.

In the first dual-treatment experiment using phosphate-buffered sucrose as the sachet medium for clone OUR, the mean number of aphids settled on the sachets or not settled differed significantly in the 25 (*F* = 55.71, df = 2, *P* < 0.0001), 50 (*F* = 33.12, df = 2, *P* < 0.0000), and 100 (*F* = 54.47, df = 2, *P* < 0.0000) μg/ml PLRV tests after 24 h, but these differences were due wholly to those aphids not settled on any sachet treatment (Table 3). Results were similar for clone BP23 in the 25 (*F* = 43.03, df = 2, *P* < 0.0001), 50 (*F* = 82.96, df = 2, *P* < 0.0001), and the 100 (*F* = 39.45, df = 2, *P* < 0.0001) μg/ml PLRV tests (Table 3).

**Table 3:**
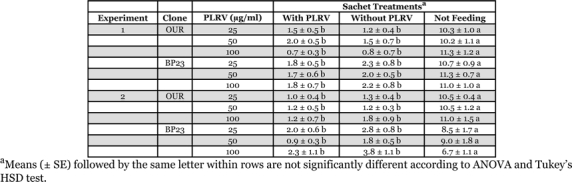
Arrestment of Myzus persicae on Parafilm^®^ membrane sachets containing phosphate-buffered sucrose with and without purified Potato leafroll virus. The experiments were conducted as dual-treatment (two sachets) tests with one sachet containing the respective amount of virus and the other containing only sucrose.

In the second dual-treatment experiment using phosphate-buffered sucrose, the mean number of clone OUR aphids settled on the sachets or not settled differed significantly in the 25 (*F* = 155.39, df = 2, *P* < 0.0001), 50 (*F* = 47.37, df = 2, *P* < 0.0001), and 100 (*F* = 24.34, df = 2, *P* < 0.0001) μg/ml PLRV tests after 24 h, but these differences were due wholly to those aphids not settled on any sachet treatment (Table 3). Results were similar for clone BP23 in the 25 (*F* = 16.77, df = 2, *P* = 0.0001), 50 (*F* = 24.70, df = 2, *P* < 0.0001), and the 100 (*F* = 39.45, df = 2, *P* < 0.0001) μg/ml PLRV tests (Table 3).

**Figure 2:**
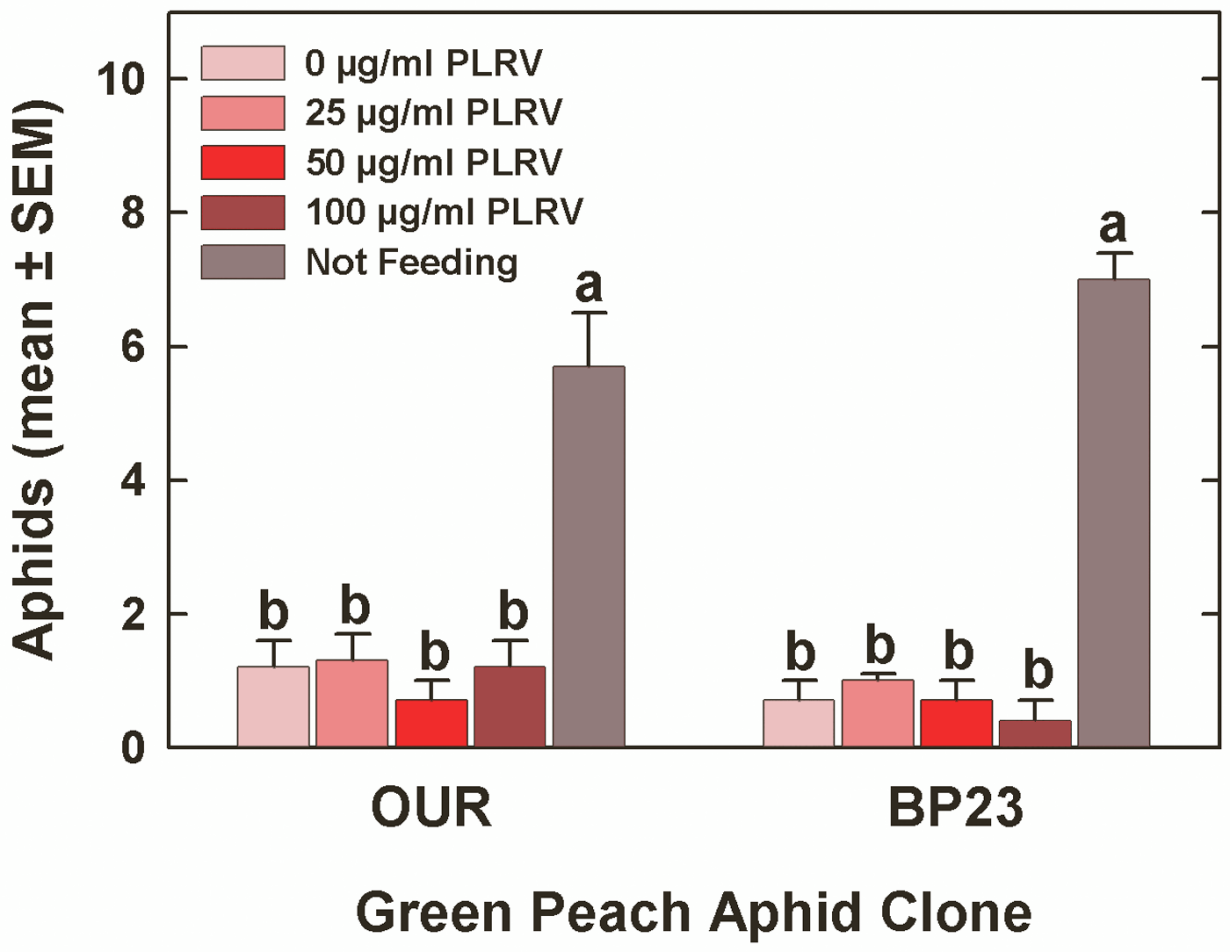
Arrestment of Myzus persicae on Parafilm^®^ membrane sachets containing phosphate-buffered sucrose with various concentrations of purified Potato leafroll virus. The experiment was conducted as a multiple-treatment test with all virus concentrations presented simultaneously. Bars within clone having the same letter are not significantly different according to ANOVA and Tukey’s HSD test.

In the multiple-treatment experiment with phosphate-buffered sucrose as the sachet medium, the mean number of aphids settled on the sachets or not settled differed significantly in the test with clone OUR (*F* = 14.02, df = 4, *P* < 0.0001) and in the test with clone BP23 (*F* = 88.48, df = 4, *P* < 0.0001) after 24 h (Figure 2). Tukey’s HSD test revealed that the significance found in the ANOVAs was attributable to those aphids not settled and there were no significant differences between any sachet treatments.

**Figure 3:**
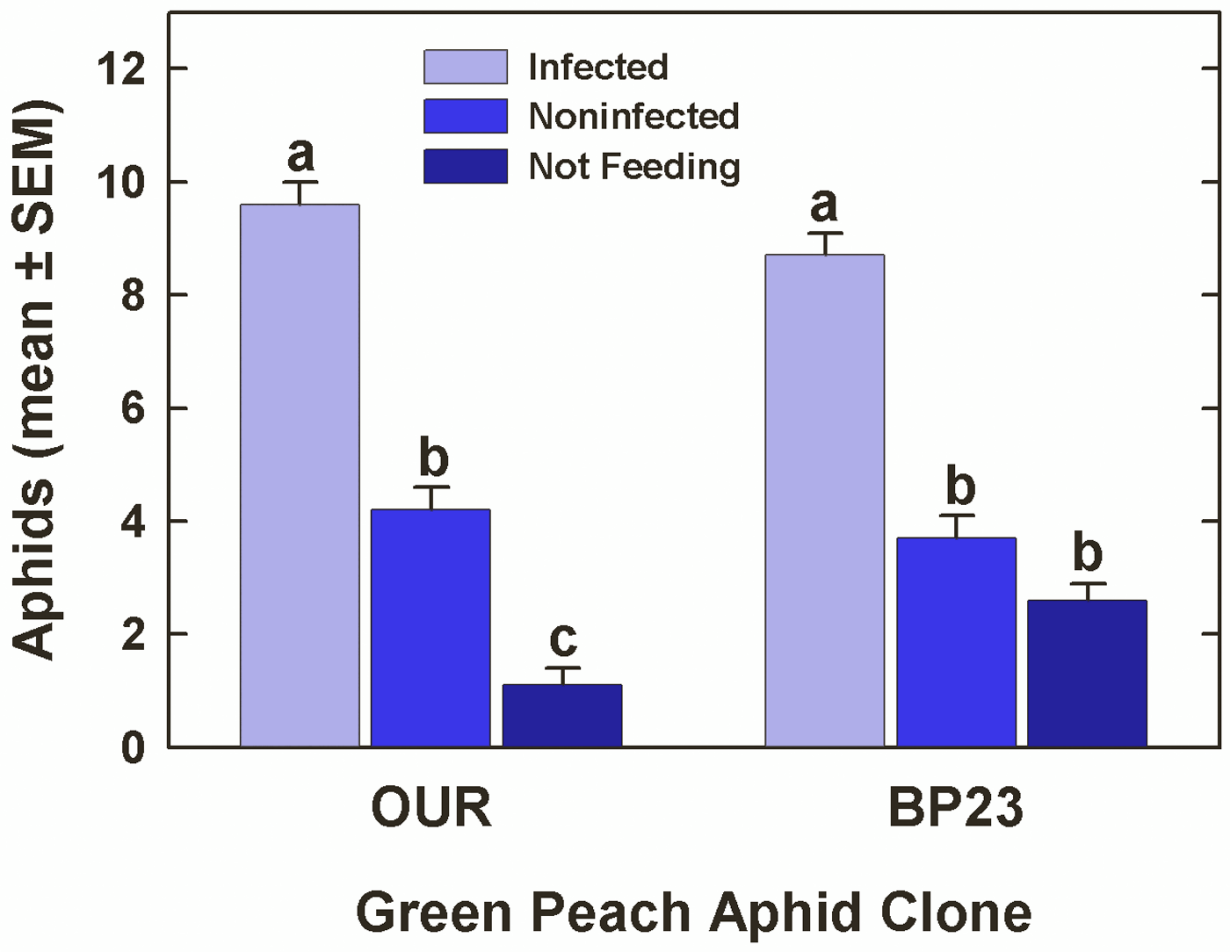
Arrestment of Myzus persicae on Physalis floridana L. leaves either infected with Potato leafroll virus or noninfected. The experiment was conducted as a dual-treatment test with both leaves presented simultaneously. Bars within clone having the same letter are not significantly different according to ANOVA and Tukey’s HSD test.

In the experiment in which excised, PLRV-infected P. floridana leaves served as the virus source for both clones, the mean number of aphids settled on the treatment leaves or not settled differed significantly in the test with clone OUR (*F* = 103.24, df = 2, *P* < 0.0001) and in the test with clone BP23 (*F* = 73.94, df = 2, *P* < 0.0001) after 24 h (Figure 3). Tukey’s HSD test revealed that significantly more aphids were settled on the PLRV-infected leaves in the clone OUR test (*P* < 0.0001) and in the clone BP23 test (*P* < 0.0001).

## Discussion

Taken as a whole, results from all the membrane sachet experiments indicate that direct encounter with PLRV does not stimulate differential behavior in M. persicae in terms of arrestment or increased settling in locations where virus is present. This is true for those experiments using MP148 diet as the sachet medium, despite significantly more aphids on two virus treatments in the first dual-treatment experiment (Table 1). In the absence of any apparent stimulatory effect by PLRV, these two results were chance events as the sachet treatments in all experiments (36 tests involving two M. persicae clones) were essentially equal.

In the process of viral acquisition, it is likely that PLRV binds to midgut receptors intended for other purposes, possibly nutrient uptake (Gildow 1993). In such a case, the results presented here indicate that midgut receptors that bind PLRV might not bind nutrients that are also phagostimulatory, such as methionine or sucrose (Dadd and Krieger 1968;Harrewijn 1983;Klinghauf 1987;Srivastava 1987), as there was no evidence of more aphids feeding on virus-containing sachets. Of course, constituents contained in MP148 diet may have masked any PLRV-related stimuli, but results from experiments using phosphate-buffered sucrose confirmed that PLRV has no arrestment effects on M. persicae. Moreover, others have used MP148 diet as a medium for PLRV acquisition by M. persicae precisely because it enhances aphid feeding and viral acquisition (van den Heuvel et al. 1991), rather than interfering with virus-vector interactions.

In the excised-leaf experiment, more than twice as many aphids from both clones had settled on PLRV-infected leaves than on noninfected leaves after 24 h. These results are in agreement with those of others dealing with the interaction of virus-infected host plants and M. persicae (van den Heuvel and Peters 1990;Castle and Berger, 1993). Compared with the results from the sachet experiments, it is apparent that M. persicae can be attracted to and/or arrested on plants infected with PLRV and do not require visual cues resulting from disease symptoms, in spite of the complex transmission relationship between virus and vector. This supports the notion that the enhanced nutritional quality of virus-infected host plants is a major factor in the interaction of PLRV and M. persicae. However, recent evidence suggests that arrestment (and possibly attraction) of M. persicae on PLRV-infected plants may have an olfactory component (Castle and Berger 1993; Eigenbrode et al. 2002), which may prove to be as important, or more so, than host plant nutritional quality in the epidemiology of PLRV.
